# CopyRighter: a rapid tool for improving the accuracy of microbial community profiles through lineage-specific gene copy number correction

**DOI:** 10.1186/2049-2618-2-11

**Published:** 2014-04-07

**Authors:** Florent E Angly, Paul G Dennis, Adam Skarshewski, Inka Vanwonterghem, Philip Hugenholtz, Gene W Tyson

**Affiliations:** 1 Australian Centre for Ecogenomics/School of Chemistry and Molecular Biosciences/The University of Queensland, St Lucia, Brisbane, QLD 4072, Australia; 2 Current affiliation: School of Agriculture and Food Sciences, Level 3, Hartley Teakle Building (83), The University of Queensland, St Lucia, Brisbane, QLD 4072, Australia; 3 Advanced Water Management Center, Level 4, Gehrmann Laboratories Building (60), The University of Queensland, St Lucia, Brisbane, QLD 4072, Australia

## Abstract

**Background:**

Culture-independent molecular surveys targeting conserved marker genes, most notably 16S rRNA, to assess microbial diversity remain semi-quantitative due to variations in the number of gene copies between species.

**Results:**

Based on 2,900 sequenced reference genomes, we show that 16S rRNA gene copy number (GCN) is strongly linked to microbial phylogenetic taxonomy, potentially under-representing Archaea in amplicon microbial profiles. Using this relationship, we inferred the GCN of all bacterial and archaeal lineages in the Greengenes database within a phylogenetic framework. We created CopyRighter, new software which uses these estimates to correct 16S rRNA amplicon microbial profiles and associated quantitative (q)PCR total abundance. CopyRighter parses microbial profiles and, because GCN estimates are pre-computed for all taxa in the reference taxonomy, rapidly corrects GCN bias. Software validation with *in silico* and *in vitro* mock communities indicated that GCN correction results in more accurate estimates of microbial relative abundance and improves the agreement between metagenomic and amplicon profiles. Analyses of human-associated and anaerobic digester microbiomes illustrate that correction makes tangible changes to estimates of qPCR total abundance, α and β diversity, and can significantly change biological interpretation. For example, human gut microbiomes from twins were reclassified into three rather than two enterotypes after GCN correction.

**Conclusions:**

The CopyRighter bioinformatic tools permits rapid correction of GCN in microbial surveys, resulting in improved estimates of microbial abundance, α and β diversity.

## Background

The advent of high-throughput sequencing has accelerated the study of natural microbial communities. Many microbial surveys rely on the sequencing of the small subunit rRNA (16S or 18S rRNA) gene. However, the analysis of microbial community structure using this molecular technique is considered semi-quantitative because methodological and biological biases can skew estimation of species relative abundance in a community. For example, the choice of DNA extraction method and PCR primers significantly affects operational taxonomic unit (OTU) representation in amplicon community profiles [1-3]. The most well known biological bias in such profiles is variation in gene copy number (GCN) between species [4]. Note that GCN refers here specifically to the copy number of the 16S rRNA gene, unless otherwise indicated.

GCN variation spans over an order of magnitude, from 1 to 15 in Bacteria, but only up to 5 in Archaea [5]. This order of magnitude range biases both amplicon microbial profiles and estimates of total microbial abundance based on amplicon quantitative PCR (qPCR) data [6]. It could be corrected by weighting read counts for a given species by the inverse of its GCN [2,4,7,8], but information about GCN is lacking for most microbial species. Since related species have similar GCN [8,9], it is often possible to accurately estimate GCN of an uncultured organism if a closely related sequenced relative exists [9,10], though this means dramatically reducing the search space to the subset of species with documented GCN. Another possibility is to place reads on a phylogenetic tree and calculate GCN based on that of sequenced relatives using phylogenetically independent contrasts (PIC) [9,11,12]. This method has the advantage of not restricting search space, but its implementation is computationally intensive [9]. Ultimately, correcting for GCN bias is still an open problem that no readily available software adequately addresses.

GCN bias limits our ability to produce accurate microbial profiles and compromises efforts that rely on relative or absolute abundance, such as the comparison of microbiomes [13], or the development of predictive models [14]. The effect of biases such as GCN may readily be apparent through the discrepancies noted in human microbiome studies using different interrogation techniques [15-17], despite the deployment of standard operating procedures [18,19]. Here we introduce CopyRighter, a new method and easy-to-use software to correct GCN bias in amplicon and qPCR studies. We test this software using mock read datasets and illustrate the effects of correction on human gut and bioreactor-associated microbial communities.

## Methods

### Variation in gene copy number within species

As a pre-requisite for curating the GCN in the Integrated Microbial Genomes (IMG) system, we investigated the natural variation in GCN between strains of the same species. We used the curated GCN entries in the Ribosomal RNA Database (rrnDB) [20], which included 153 bacterial and archaeal species containing 2 to 40 strains. The difference (*d*) between the mean (*x*) and extremum (maximum or minimum) GCN for these species was calculated and plotted. Except for a single species (*Bifidobacterium animalis*), this difference generally had the upper bound: *d* ≤ 0.105 *x* + 0.720 (Additional file 1: Figure S1).

### Calculation of gene copy number in sequenced genomes

The CGN was inferred from 4,512 sequenced microbial genomes downloaded from IMG version 4.0, released in October 2012. Though GCN is reported by IMG, errors in the GCN records required us to perform manual curation. RNAmmer 1.2 [21] and INFERNAL 1.1rc1 [22] were run independently to estimate the GCN of these genomes. The GCN of a particular genome was considered suspicious if: 1) it was smaller than 1 or larger than 15; 2) the average contig length was smaller than 200 kbp; 3) it was not identical to that predicted by INFERNAL or RNAmmer; or 4) it differed significantly from IMG's or rrnDB's average for this species (>1.2 f(*x*); Additional file 1: Figure S1). A resolution of suspicious GCN was attempted by ignoring the IMG record and: 1) using the GCN determined by INFERNAL or RNAmmer if it was consistent with rrnDB; 2) using the value from INFERNAL or RNAmmer if this genome was not represented in rrnDB but its scaffold length was longer than 200 kbp; and 3) using IMG's 5S or 23S rRNA GCN if it agreed with rrnDB's GCN (when IMG's 16S rRNA GCN was zero). This correction was repeated as necessary after removing potentially truncated 16S rRNA genes (<1,220 bp). Suspicious GCNs that could not be corrected were removed from subsequent analyses. This procedure detected 278 suspicious values, 259 of which could be corrected, resulting in GCN values for 2,982 genomes. This analysis can be reproduced using the CopyRighter preprocessing scripts available at http://github.com/fangly/AmpliCopyrighter/releases.

### Reconstruction of the gene copy number of microbial taxa

We estimated the GCN of archaeal and bacterial taxa in Greengenes from October 2012 [23]. First, text searches were performed to match each IMG genome name to a Greengenes species name and ID. These IDs were then replaced by the ID of their representative sequence from the Greengenes file of OTUs clustered at 99% identity. This allowed us to place each genome and its GCN on the Greengenes phylogenetic tree (with OTUs clustered at 99% identity) and prune the tree. For genomes matching to the same Greengenes OTU, an average of their GCN was calculated. Estimates of GCN for each ancestral node in the tree were calculated using the PIC method [11], which essentially combines the GCN of sequenced daughter species on the tree linearly based on their phylogenetic distance. However, several nodes in a tree can belong to the same taxon. To accommodate for this, the GCN of a taxon was calculated as the weighted average of the GCN for the corresponding nodes, with the weight being proportional to the number of nodes making up the GCN estimate. The results were GCN estimates plotted from species to phylum level on the Greengenes taxonomy. This method was implemented using the Bio::Phylo Perl modules [24] and Newick Utilities [25], and can be run using the scripts available from http://github.com/fangly/AmpliCopyrighter/releases.

### Phylogenetic and taxonomic signal of microbial gene copy number

We built a tree based on the Greengenes taxonomy, using an arbitrary branch length of 1.0, and attached the empirical GCNs to the corresponding taxa. The tree was parsed and pruned with the APE [26] and Picante [27] R libraries, and the Phytools library [28] computed the λ statistic [29], which expresses the strength of phylogenetic signal. The value of λ ranges from 0, representing an absence of link between a trait and a phylogenetic tree, to 1, indicating a strong link. A *P* value was calculated from a likelihood ratio test against the null hypothesis that λ is 0. This statistic was calculated using the Greengenes taxonomic tree constructed above and repeated using the Greengenes phylogenetic tree.

### Estimation of microbial gene copy number for unsequenced species

**Figure 1:**
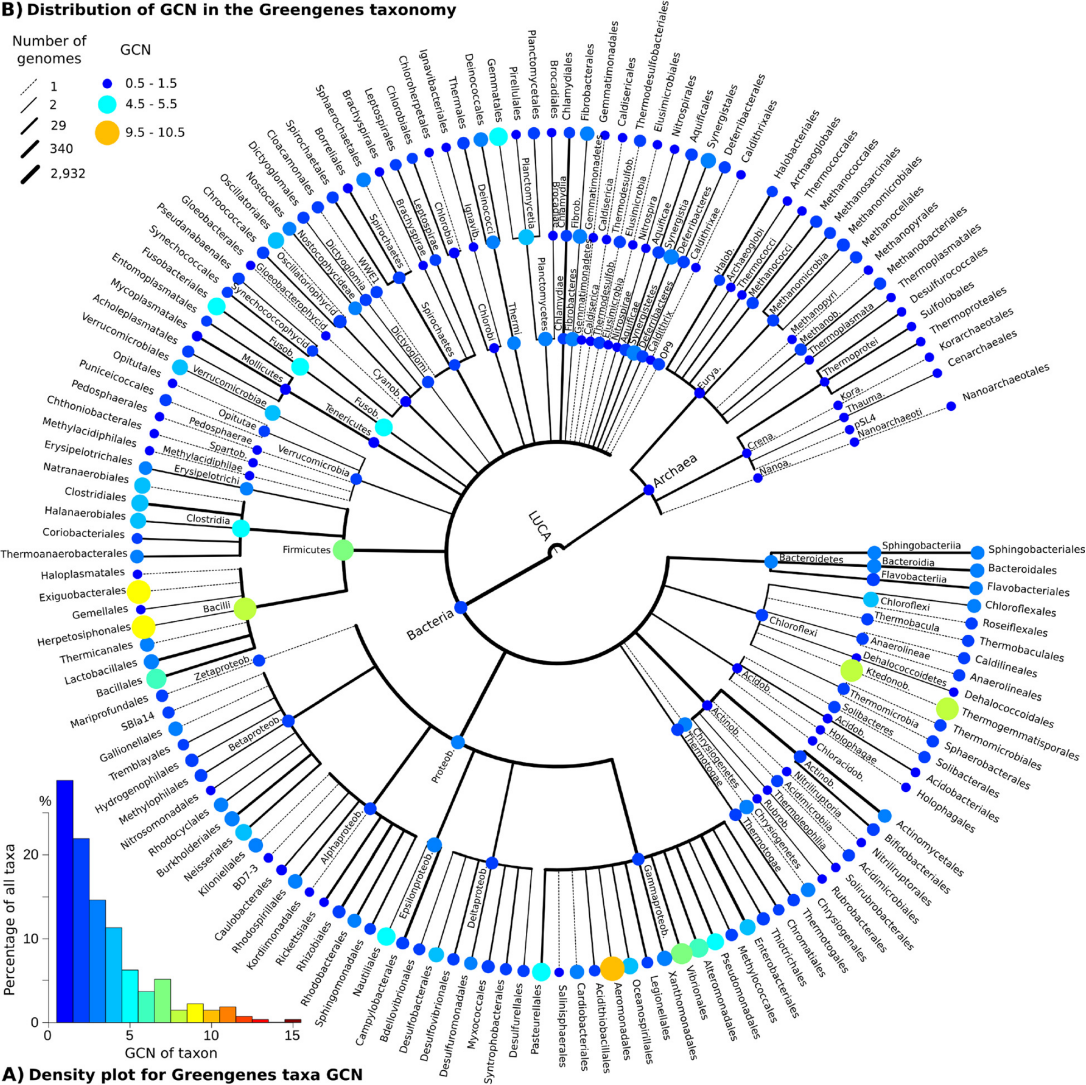
**CopyRighter flowchart for the correction of microbial amplicon datasets. (A)** Pre-computation of genome copy number (GCN) based on a tree-based taxonomy and reference genomes. **(B)** The processing of microbial data through off-the-shelf programs. **(C)** The correction of microbial data to estimate relative abundance, absolute abundance and average GCN in given samples. OTU, operational taxonomic unit; qPCR, quantitative polymerase chain reaction; rRNA, ribosomal RNA.

The PIC method was combined with rerooting [12] to estimate the GCN of unsequenced species (Figure 1A). These estimates were then mapped on the Greengenes taxonomy. Specifically, when several tree nodes mapped to a single taxon, estimated GCNs were removed if empirical values were present, and the mean of the remaining values was calculated. This intensive computation was performed for all 177,814 Greengenes records on the tree on 48 to 64 core high-performance computers, resulting in a table of pre-computed GCNs for all Greengenes OTUs and taxa. The scripts written for this step used the Newick Utilities [25] for quick processing of the Newick-formatted tree and can be downloaded from http://github.com/fangly/AmpliCopyrighter/releases.

### Correction algorithm

We implemented a Perl program called CopyRighter, that uses modules from Bio-Community (http://search.cpan.org/dist/Bio-Community/) [30] and systematically corrects 16S rRNA gene amplicon datasets by taking into account the varying GCN in microbial species (Figure 1C). CopyRighter is available under the GNU General Public Licence v3.0 at http://github.com/fangly/AmpliCopyrighter/releases. CopyRighter reads OTU tables (in tabular, biom, QIIME, GAAS or UniFrac format) that contain Greengenes taxonomic assignments. For each OTU, CopyRighter looks up its estimated GCN from the pre-computed table described above and weights the number of 16S rRNA gene amplicon reads of this OTU by th e): $r_{i}=\frac{c_{i}}{g_{i}}\times \frac{100}{\sum_{j}^{S}\frac{c_{j}}{g_{j}}}$, where *r*_i_ is the relative abundance of OTU *i*, *c*_*i*_ its count, *g*_*i*_ its GCN and *S* is the community richness. OTUs without any taxonomic assignment are assigned a GCN equal to the average value in the community to prevent them from affecting the relative abundance of other OTUs. The results are saved in a new account file in the same format as the input file and the average GCN for each community is returned. Provided qPCR results in a tab-delimited text format, CopyRighter corrects qPCR numbers by dividing them by the GCN averages in the corresponding communities.

### Validation using *in silico* mock communities

To assess the accuracy of CopyRighter, we simulated 90 microbial communities, divided into low, medium and high richness groups (10,100 and 1,000 species, respectively) using Grinder [31]. All communities were designed with a power law rank-abundance structure [32], the most abundant species representing 20% of the community (Additional file 2: Figure S2). For each of the 30 replicates per group, Grinder took IMG genomes and assigned them a random abundance rank. These artificial communities were sequenced *in silico* by Grinder using the Roche-454 GS-FLX Ti technology routinely used in microbial surveys. Each community was sequenced *in silico* twice, once using the 16S rRNA gene amplicon approach (universal primers pyroLSSU926F AAACTYAAAKGAATTGRCGG and pyrolSSU1392R ACGGGCGGTGTGTRC, targeting gene hypervariable regions V6-V8), and once using a shotgun metagenomic approach.

### Validation using *in vitro* mock datasets

To further validate CopyRighter, we used published cell-based and DNA-based *in vitro* mock datasets. The mock 16S rRNA gene amplicon dataset from Yuan and colleagues [2] was produced by pooling an equal number of cells from 11 microbial species commonly found in the human body, prior to DNA extraction and amplicon sequencing with universal primers specific to the V1-V2 hypervariable regions (cell-based mock). We also used a V3-V5 16S rRNA gene amplicon dataset from the Human Microbiome Project (accession SRR074387) [18,33], which was generated by extracting the DNA from 22 microbial species separately, and pooling their genomic DNA in the ratio needed to obtain an equal number of 16S rRNA gene copies for each species (DNA-based mock). The cell-based and DNA-based *in vitro* mock datasets were sequenced using a Roche-454 GS-FLX Ti sequencer [2,33]. We processed these data bioinformatically using the 16S rRNA gene amplicon protocol described below.

### Processing of 16S rRNA gene amplicon read datasets

An OTU clustering and sequence-similarity taxonomic annotation approach was used to process 16S rRNA gene amplicon datasets. Reads from distinct samples were first separated according to their multiplex identifier using QIIME [34] and their 454 sequencing errors were corrected using Acacia [35]. Sequences were trimmed to recover the mode of the length distribution (that is, 300 bp generally). CD-HIT-OTU [36] was used to remove chimeras and cluster reads into 97% identity OTUs, which were then given a taxonomic affiliation by performing a BLASTN search [37] against the Greengenes database. The resulting OTU table was rarefied to 1,000 reads (when possible) using at least 100 repetitions, and summarized at the genus level using Bio-Community scripts.

### Post-processing of mock datasets

We calculated the expected species relative abundance for each *in vitro* mock community. For cell-based mocks, the number of cells for each species was simply normalized to 100%. For DNA-based mocks, the total number of 16S rRNA gene templates added in the DNA pool for each species was divided by the GCN of the corresponding genome, and normalized to 100%. A pipeline was setup with Bpipe [38] to process the result of 16S rRNA gene amplicon datasets. Each dataset was processed by converting read counts into relative abundance (no correction) and correcting GCN at the phylogenetic and taxonomic level. The taxonomic string of the OTUs in the sampled community was corrected when inspection revealed an obvious difference in assignment compared to the sample communities. The data were Hellinger-transformed [39] normalized to 100% and the euclidean distance between the resulting sample community composition and the expected species relative abundance was calculated for each sample. A unilateral exact Mann–Whitney test was performed (wilcox.test function in R) to estimate if the distances between corrected and expected community profiles were significantly lower than the distances between non-corrected and expected profiles.

### Re-analysis of twin gut cohort microbiomes

A cohort of twins and their mother has been previously followed using V1-V2 16S rRNA gene amplicon pyrosequencing to investigate the composition of their gut microbiota [40,41]. We re-analyzed the 280 microbiomes with at least 1,000 reads through the pipeline described above, using a trimming threshold of 200 bp, and corrected for GCN using CopyRighter at the phylogenetic level. A first analysis consisted of summarizing the microbial data at the phylum level and calculating the Berger-Parker α diversity index in each sample. Starting with the samples with the most similar Berger-Parker index, a bilateral Mann–Whitney test was run with an increasing number of samples to determine the fraction of non-significantly different samples before and after correction. A second analysis was performed to analyze the effect of correction on sample β diversity. We classified the data into enterotypes in R, as described previously [15]. The Bray-Curtis distance was calculated using the Vegan library [42], partition around medoids clustering was performed using the FPC library and the average silhouette width and Calinski-Harabasz index were recorded. In addition, the indicator value of each genus was calculated to identify potential indicator genera using the R Indicspecies library [43].

### Analysis of microbial abundance in anaerobic digesters

Replicate bioreactors were operated for 362 days to obtain information about the total microbial abundance (16S rRNA qPCR) and composition of microbial communities (16S rRNA gene amplicon pyrosequencing reads) (primers pyroLSSU926F and pyrolSSU1392R, targeting region V6-V8) involved in the anaerobic digestion process (Additional file 3: Supplementary Protocol). The 16S rRNA gene amplicon reads (deposited under accession number SRR1145444) were processed using the bioinformatic pipeline described above, using 800 reads for the rarefaction step, and corrected for GCN with CopyRighter at the phylogenetic level to obtain estimates of relative abundance. qPCR results were also corrected by CopyRighter to compare the microbial abundance (number of genomes/ml of extracted reactor fluid) between sampling dates. The results were summarized at the order level and plotted. Unilateral paired t-tests were performed (t.test function in R) to determine if the total abundance estimates from day 362 were lower than those for day 27.

## Results

### Gene copy number taxonomic signal

**Figure 2:**
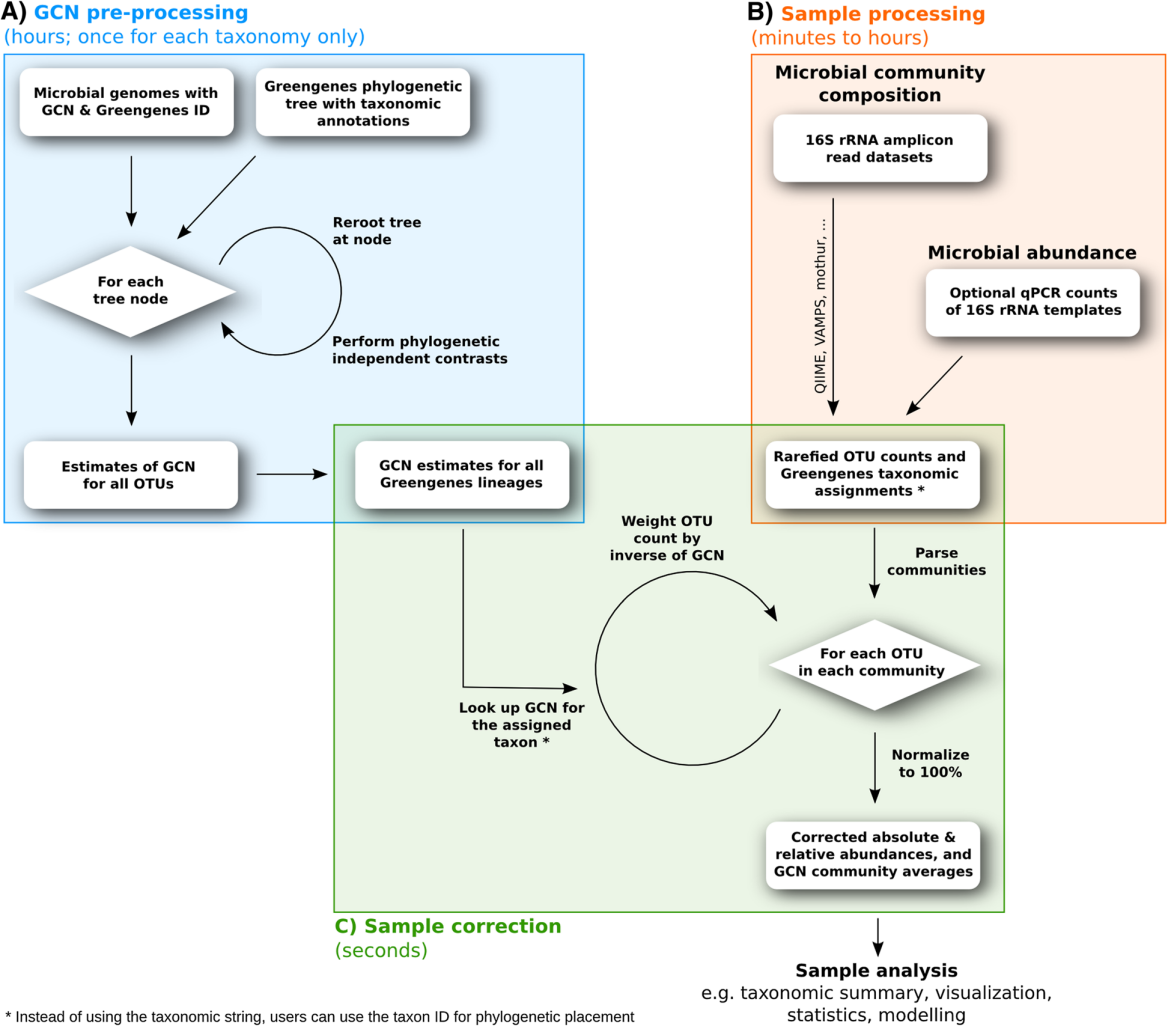
**Estimated gene copy number (GCN) of 274 Greengenes taxa represented by over 2,900 genomes. (A)** Density plot of GCN. **(B)** Distribution of GCN from phylum to order level.

Greengenes provides a phylogenetic tree based on bacterial and archaeal rRNA sequences, and a taxonomic system derived from this tree [23]. We calculated the GCN from over 2,900 sequenced microbial genomes from the IMG database [44] using RNAmmer and INFERNAL, and averaged these values for each of the 274 Greengenes taxa represented by multiple genomes. GCN ranged from 1 for most Greengenes taxa (28.8%) to 15 copies for a single species, *Photobacterium profundum* (Figure 2A).

Using the PIC method for reconstruction of ancestral traits [11], we estimated the GCN for all Greengenes taxa (Figure 2B). Variations between taxa were present at different taxonomic levels. For example, the estimated GCN for the Firmicutes (6.81) and Fusobacteria (4.81) were well above that of other phyla. Similarly, at the domain level, Bacteria (2.40) had a higher GCN than Archaea (1.46).

We calculated the *λ* statistic of phylogenetic signal [29] for empirical GCN using the Greengenes phylogenetic tree and found that GCN is correlated with phylogeny (*λ* = 0.844, *P* = 2.13e-176). This association was also apparent when using the Greengenes taxonomy which is a simplified and less resolved representation of the phylogeny (*λ* = 0.546, *P* = 2.03e-38). This suggests that GCN can be reliably inferred from microbial phylogeny and tree-based taxonomy.

### CopyRighter

We have implemented a program called CopyRighter that takes GCN into account when estimating OTU relative abundance. CopyRighter is freely available under the GNU General Public License v3.0 from http://github.com/fangly/AmpliCopyrighter/releases. Exploiting the strong phylogenetic and taxonomic signal in GCN, we used PIC with rerooting [12] to compute estimates of GCN for the 1.08 million records in Greengenes based on publicly available GCN information from 2,900 reference genomes (Figure 1A) (data available at http://github.com/fangly/AmpliCopyrighter/releases). CopyRighter corrects the GCN bias for each OTU in a microbial dataset using these estimates (Figure 1C). Additionally, given qPCR assay data, CopyRighter divides the number of 16S rRNA gene templates [45] by the calculated average GCN in the community to estimate total microbial abundance in a sample.

Correcting microbial datasets with CopyRighter requires executing a single command after any analysis pipeline that produces community profiles (file of rarefied OTU counts, in tabular, biom, QIIME, GAAS or UniFrac format) containing Greengenes taxonomic assignments (Figure 1B). When correcting, CopyRighter can use two methods to find the GCN of an OTU: 1) based on the location of its assigned taxon on the Greengenes phylogenetic tree (identified by its taxon ID) (phylogenetic-level correction); or 2) based on the Greengenes taxonomic string of this taxon (taxonomic-level correction). The output of CopyRighter is a corrected file expressing OTU relative abundance (as a percentage), GCN averages for each community and optional corrected qPCR results. For ease-of-use, we created a Galaxy front-end [46], available at http://toolshed.g2.bx.psu.edu/view/fangly/copyrighter.

### Software validation

**Figure 3:**
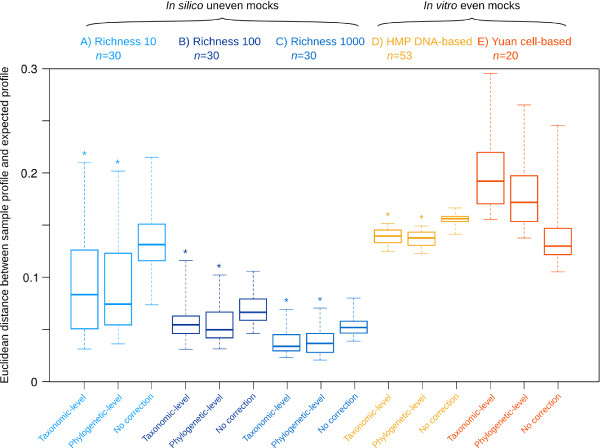
**Boxplot of the accuracy of CopyRighter's correction based on the composition of 16S rRNA gene amplicon mock datasets at the genus level. (A-C)***In silico* uneven Grinder datasets of varying richness, and **(D,E)** published *in vitro* mock datasets. The boxes represent the minimum, maximum, median and interquartile range. The smaller the distance, the closer the observed profile is to the expected profile. Corrected profiles with a significantly lower distance than the corresponding uncorrected profiles (unilateral exact Mann–Whitney test, *P* < 0.05) are marked with a star.

To assess the accuracy of CopyRighter, we generated 90 microbial communities *in silico* and simulated their 16S rRNA gene amplicon and metagenomic shotgun sequencing using Grinder [31]. The distances between observed and expected amplicon microbial profiles were significantly smaller for GCN-corrected than for uncorrected samples (Figure 3A-C). Phylogenetic-level correction was generally slightly more accurate than taxonomic-level correction. On average, distance decreased with the richness of the mock communities tested, from 10 to 1,000 species (Figure 3A-C). However, CopyRighter correction was beneficial across this entire richness range, with a smaller distance for corrected than uncorrected profiles (Figure 3A-C).

To complement the results from *in silico* mock communities, more realistic but low richness *in vitro* mock communities were corrected in the same manner (Figure 3D,E). Again, CopyRighter correction resulted in smaller distances between observed and expected profiles for the DNA-based mock (Figure 3D), except in the case of the cell-based mock community (Figure 3E).

Assuming that GCN correction is effective, we would expect microbial profiles obtained from different methods (for example, amplicon and metagenomic sequencing) to be more similar after CopyRighter correction. When comparing the profile of 16S rRNA *in silico* mocks to the whole genome-based profile of the corresponding metagenomic mock, we noted that GCN-corrected profiles had a significantly smaller distance than non-corrected profiles (Additional file 4: Figure S3).

### Correction of human gut microbial profiles

**Figure 4:**
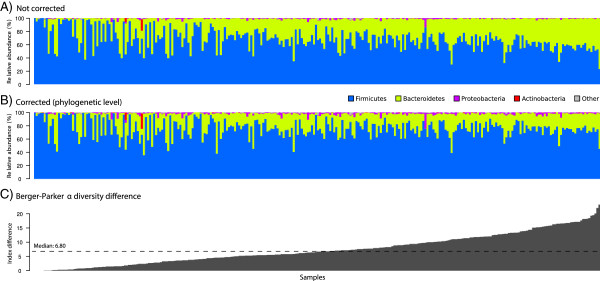
**Phylum-level effects of gene copy number bias correction in 280 human gut microbiomes. (A)** Uncorrected, **(B)** after phylogenetic-level correction, and **(C)** difference in Berger-Parker α diversity index between the corrected and non-corrected samples. Samples in all panels were sorted by increasing Berger-Parker difference.

To evaluate the impact of CopyRighter on the interpretation of empirical datasets, we corrected GCN in human gut microbiome profiles from lean and obese twins and their mothers (153 individuals) [40,41]. The correction led to phylum-level changes in relative abundance, with an overall decrease of Bacteroidetes from a median of 31.1 to 21.7% and an increase of Firmicutes from a median of 67.1 to 76.0% (Figure 4A and B). Microbiome α diversity was measured using the Berger-Parker index (that is, the relative abundance of the most abundant phylum). The difference in Berger-Parker index between corrected and non-corrected samples ranged from 0 to 23.3% (Figure 4C). Up to 53.6% of the samples did not have a statistically different Berger-Parker index (bilateral Mann–Whitney test, *P* < 0.05; Additional file 5: Figure S4).

**Figure 5:**
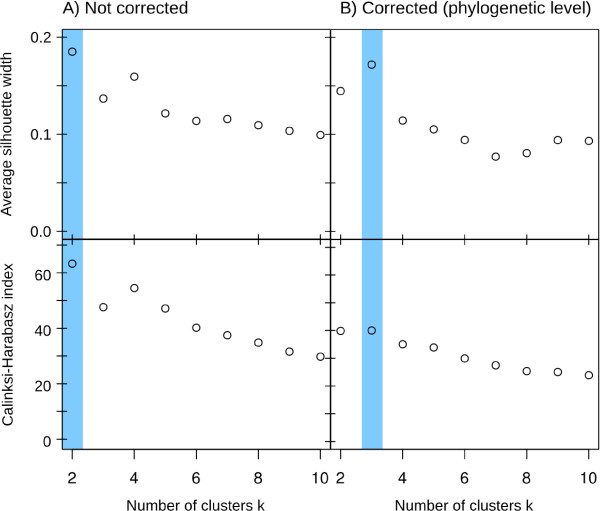
**Optimal number of enterotypes based on partition around medoid clustering of microbial profiles of the twin cohort at the genus level. (A)** Non-corrected samples, and **(B)** samples processed through CopyRighter. The optimal number of enterotypes is shaded and represents the number of clusters with the largest average silhouette width (top panels) and Calinksi-Harabasz index (bottom panels).

We calculated the Bray-Curtis distances between all gut microbiomes and clustered them using partition around medoid to determine enterotypes, as previously described [15]. The microbiomes initially clustered into two enterotypes (Figure 5A), but correcting for GCN bias indicates that the data were better partitioned into three enterotypes (Figure 5B; Additional file 6: Figure S5B). As a consequence, 23.9% of the samples would have been misclassified without GCN correction (Additional file 6: Figure S5A,B). In terms of β diversity (Additional file 6: Figure S5C), sample separation was driven mainly by the family Bacteroidaceae (genus *Bacteroides*) for enterotype A', the families Lachnospiraceae (genera *Ruminococcus*, *Roseburia*, *Blautia* and *Coprococcus*) and Coprobacillaceae for enterotype B', and the families Prevotellaceae (genus *Prevotella*) and Ruminococcaceae (genera *Ruminococcus*, *Faecalibacterium* and *Oscillospira*) for enterotype C'. Among these taxa, *Prevotella* was the only indicator species (indicator value of 0.785 for enterotype C', *P* = 1e-4).

### Correction of absolute microbial abundance

**Figure 6:**
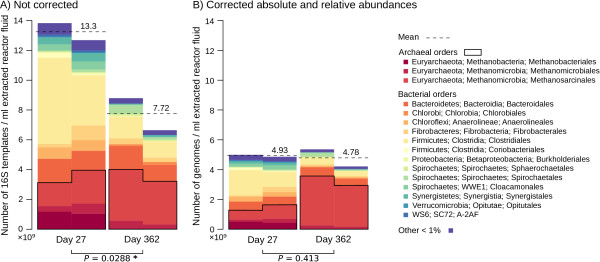
**Abundance of microbial orders in replicate anaerobic digesters. (A)** Before and **(B)** after phylogenetic-level correction of relative and total abundance. *P* values from unilateral paired t-tests are indicated, and marked with a star when significant (*P* < 0.05).

To assess the effects of GCN correction on the estimated absolute abundance of microorganisms, we analyzed samples from two replicate anaerobic digester bioreactors. For each time point, total abundance was inferred from the number of 16S rRNA gene templates determined by qPCR and microbial profiles were generated based on 16S rRNA gene amplicon reads. Copyrighter reported an average GCN of 2.69 for the samples collected on day 27, and 1.61 for the day 362 samples. This was primarily due to a change in the ratio of Bacteria (mostly represented by Clostridiales) to Archaea (mostly Methanosarcinales) over the two time points; 2.75 to 1.11 in uncorrected data, and from 2.33 to 0.460 in corrected data. Uncorrected qPCR results (Figure 6A) indicated a significant biomass decrease from 13.3 to 7.72 × 10^9^ genomes/ml extracted reactor fluid during the operation of the reactors (paired t-test, *P* = 0.0288), while corrected numbers (Figure 6B) were not different (paired t-test, *P* = 0.413), averaging 4.86 × 10^9^ genomes/ml over the two time points.

## Discussion

### Gene copy number is linked to tree-based taxonomy

We determined the 16S rRNA GCN of over 2,900 sequenced genomes and assigned these values to 274 unique taxonomic locations in the Greengenes phylogenetic framework (Figure 2A). We found that one-third of these taxa have a GCN of 1 (that is, a single 16S rRNA gene), in apparent contrast with previous reports of a modal GCN of two gene copies per genome [5,10]. This difference between GCN per taxon (that is, phylogenetically normalized) and per genome reflects that a limited number of high-GCN taxa of medical or biotechnological interest have been the subject of extensive research and sequenced many times. For example, IMG contains 116, 78 and 47 genomes of *Escherichia coli*, *Staphylococcus aureus* and *Bacillus aureus*, respectively, species that all have a GCN higher than 5.

We identified large differences in GCN between taxa at different taxonomic levels (Figure 2B). For example, bacterial taxa appear to have an additional 16S rRNA gene on average compared to archaeal taxa. Hence the relative abundance of Archaea is possibly systematically underestimated in amplicon surveys, compared to that of Bacteria, a problem that may be confounded by some primer sets [47].

Based on the *λ* statistic of phylogenetic signal, we determined that the distribution of GCN in microbial genomes is not random. The value of *λ* typically varies from 0 for a lack of signal, to 1 for a strong signal. GCN was strongly correlated with the microbial phylogeny represented by the Greengenes phylogenetic tree (*λ* = 0.844), consistent with previous evidence for a phylogenetic signal in GCN [9]. GCN was also correlated with the Greengenes taxonomy (*λ* = 0.546), because this taxonomy is derived from the Greengenes phylogeny. However, the signal was weaker in the taxonomy than in the phylogeny because each Greengenes taxon may encompass multiple nodes of the phylogenetic tree and is thus less precise. Nevertheless, the presence of GCN signal in microbial phylogeny and taxonomy makes it possible to infer GCN for organisms lacking a genome sequence.

### Copyrighter is a new tool for gene copy number bias correction

GCN varies by over an order of magnitude between microbial species and, thus, a one-to-one relationship between a 16S rRNA gene amplicon read and a microbial cell cannot be assumed. Not accounting for GCN differences between species can lead to misinterpretation of 16S rRNA gene amplicon profiles [48]. We have introduced CopyRighter (Figure 1), a software tool that aims at making amplicon surveys more quantitative by accounting for GCN bias. Our software is accurate because it uses phylogenetically-informed GCN, and is fast because we pre-compute these estimates for the entire microbial tree using the PIC framework [12]. The approach and pre-computation removes the need for computationally intensive processes such as inserting sequences in a tree [9] and, as a result, is extremely fast. For example, an OTU table containing 1,000 OTUs distributed across 10 communities only takes 14 seconds to process on a personal computer (with an Intel U7300 processor running at 1.30 GHz).

### CopyRighter improves estimates of relative abundance

CopyRighter was validated with 90 *in silico* mock amplicon datasets and produced microbial profiles closer to the expected profiles than without correction (Figure 3), and also more congruent with simulated metagenomes (Additional file 4: Figure S3). These observations held true regardless of community richness, even though accuracy was seemingly decreased at lower richness levels, reflecting the fact that any potential error when estimating the relative abundance of a species has a larger effect on microbial profiles that include only few species, compared to richer communities. Correction performed at the phylogenetic level appeared slightly more accurate than at the taxonomic level, presumably a result of the higher GCN signal in the phylogeny compared to the taxonomy. GCN correction also appeared beneficial when validating CopyRighter with published DNA-based *in vitro* mock communities, but not with the cell-based *in vitro* mock community. Since correction was effective on the *in silico* and DNA-based *in vitro* mocks, this result does not invalidate the performance of CopyRighter. Instead, this shows that experimental procedures such as DNA extraction may produce a pool of genomic DNA that bears little resemblance to the original community [1,2]. In some cases, these experimental biases may be of higher magnitude than that introduced by GCN bias, such that GCN correction may exacerbate observed differences between observed and expected community profiles and appear ineffective (Additional file 7: Figure S6).

### Gene copy number correction influences α and β diversity

To evaluate the effects of CopyRighter on empirical datasets, we re-analyzed human gut microbiomes from a cohort of twins. Firmicutes and Bacteroidetes were numerically dominant, both in corrected and uncorrected profiles (Figure 4A and B), as confirmed in previous microarray and metagenomic studies [13,49]. GCN correction did, however, create large phylum-level shifts in favor of the Firmicutes in many of these datasets, resulting in significantly different Berger-Parker α diversity estimates for about half the samples (Additional file 5: Figure S4). These shifts appear counter-intuitive given the higher average GCN of the Firmicutes relative to the Bacteroidetes (6.81 versus 2.62) but can be explained by the GCN values of individual high abundance phylotypes in the samples, which are atypical for their phyla (Additional file 8: Figure S7). Considering that the ratio of bacterial phyla in the gut is linked with disorders such as obesity [50], diabetes [51] and *Clostridium difficile* infections [52], it is important to correct for GCN to fully understand the implications of the microbiota in health and disease.

We also noted that GCN correction did not have a uniform effect on related gut microbiome samples, with the Berger-Parker index changing anywhere from 0 to 23.3% between uncorrected and corrected samples (Figure 4C). In other words, even though all samples came from the same type of habitat, GCN correction made no difference for some samples and large phylum-level differences for others. Therefore it should not be assumed that the effects of GCN correction can be inferred based on habitat type; rather samples should be individually corrected to allow more robust biological interpretation.

Gut microbiomes have been classified into enterotypes based on their prevalent microbial species [13]. Our enterotype classification results were generally consistent with existing studies of the distal gut, defining two main *Bacteroides* and *Prevotella-*dominated enterotypes [15,53]. Considering that the ordination of human microbiomes can result in smooth gradients [54], the exact number of enterotypes is contested [13,15]. Though our analysis was limited to a single cohort, the microbial profiles corrected by CopyRighter support the existence of a third enterotype based around *Ruminococcus,* in accordance with a previous metagenomic study [13]. Thus, not accounting for GCN has important implications and may lead to incorrect enterotype classification.

### Gene copy number correction affects absolute microbial abundance estimates

To improve absolute microbial abundance estimates, CopyRighter can be used to correct amplicon qPCR results that have a corresponding community profile. In uncorrected 16S rRNA qPCR results from anaerobic digesters, the biomass seemed to be halved over 355 days (Figure 6A). However, corrected numbers indicate that total biomass was not significantly different between the two time points and the ratio of major functional groups was also misrepresented (Figure 6B). This may have important implications for interpreting community dynamics and function.

### Advantages and limitations of CopyRighter

Most microbial species have no genome representative and their GCN is unknown, which is an impediment to the correction of GCN in microbial datasets. In the CopyRighter approach, we have pre-computed GCN estimates for over a million Greengenes records, an advance made possible by leveraging publicly available genomes, phylogenetic trees and taxonomic systems within the context of the PIC framework. However, there are still many phyla without genome representation, especially candidate phyla [55], for which GCN estimates are likely not as accurate as those from well-represented phyla. Further, not all GCN values reported by IMG are correct, phylogenetic trees are affected by lack of representation and uncertainty [56], and the Greengenes taxonomy does not always include species information in its taxonomic strings. These drawbacks limit the precision of our pre-computed GCN estimates, but with further database growth and expert curation in time, updates to CopyRighter data files will make these problems less significant.

CopyRighter correction represents the last step of many experimental and bioinformatic steps to estimate microbial community composition. Community profiles can be seriously compromised by experimental procedures such as DNA extraction [1,2] (Figure 3E; Additional file 7: Figure S6), whole genome multiple displacement amplification [57,58], PCR [3,47] and sequencing [35,59]. In some instances, these upstream issues may be more problematic than GCN bias. Despite these potential limitations, our validation using mock datasets demonstrate that Copyrighter-based GCN correction is effective in improving the fidelity of community profiles.

While Copyrighter brings us a step closer towards estimating accurate OTU relative abundance in environmental surveys, it does not address genome copy number bias. Genome copy number varies during the natural bacterial life cycle, doubling during replication, and some endosymbiotic and thermophilic bacteria exhibit extreme polyploidy or large genome copy variations [60-62]. The magnitude and effects of genome copy number bias on biological interpretations are largely unknown and will be challenging to address in a systematic fashion.

## Conclusions

CopyRighter is a user-friendly open source software tool that enables rapid correction of GCN bias thereby improving the accuracy of amplicon-based community profiling and microbial biomass estimations. In addition, the average community GCN calculated by CopyRighter may provide insights into environmental conditions since GCN reflects the ecological strategies of microbial species, with higher average GCN in faster growing communities, in locations where resources are not limiting [63,64].

As illustrated throughout the present study, correcting for GCN is important since it can significantly alter estimated total microbial abundance, α and β diversity and, ultimately, biological interpretation. One should expect the effects of CopyRighter correction to be more pronounced when many species in a community differ strongly in GCN; for example, when a microbial profile contains many Archaea (GCN of 1.46 on average) and Firmicutes (GCN of 6.81). However, communities often contain tens to thousands of species, making prediction of the effects of correction non-trivial. In practice, the consequences of correction are different for every sample, even for samples originating from the same habitat, as seen in the survey of the twin cohort microbiota. Fortunately, CopyRighter is fast and compatible with popular taxonomy-based analysis workflows. We recommend running Copyrighter systematically on every microbial sample, if possible using phylogenetic-level rather than taxonomic-level correction, to obtain the highest accuracy possible.

We have produced freely available data files of the phylogenetically-based estimates of GCN for all OTUs and taxa in the Greengenes database. We anticipate that the CopyRighter methodology and software described here will be extended to cover GCN in other taxonomies (for example, Silva [65]), or other variable copy number genes or intergenic marker regions, or different genome characteristics altogether. For example, given appropriate pre-computed estimates (Figure 1A), CopyRighter could correct the GC percent bias introduced by sequencing microbial samples [66,67], genome length bias in metagenome profiles [68], or improve fungal surveys, in which the internal transcribed spacer sequenced can vary by two orders of magnitude [69].

## Abbreviations

bp: base pair; GCN: gene copy number; IMG: Integrated Microbial Genomes; OTU: operational taxonomic unit; PCR: polymerase chain reaction; PIC: phylogenetically independent contrasts; qPCR: quantitative polymerase chain reaction; rrnDB: Ribosomal RNA Database; rRNA: ribosomal RNA.

## Competing interests

The authors declare that they have no conflict of interest.

## Authors’ contributions

FEA wrote the software, designed the study, performed data analysis and redacted the manuscript. PGD designed the study and performed data analysis. AS wrote the software. IV operated bioreactors and processed bioreactor samples. PH and GWT designed the study and redacted the manuscript. All authors read and approved the final manuscript.

## Supplementary Material

Additional file 1: Figure S1 Variation in gene copy number between strains of the same species in the Ribosomal RNA Database. The size of the bubbles indicates the number of species represented, from 1 to 23. Click here for file

Additional file 2: Figure S2 Rank-abundance plot of the low, medium and high richness *in silico* mock communities generated with Grinder. Click here for file

Additional file 3 Supplementary protocol: operating and sampling anaerobic digesters. Click here for file

Additional file 4: Figure S3 Boxplot of the agreement between *in silico* 16S rRNA gene amplicon and metagenomic mock datasets with and without Copyrighter correction. The boxes represent the minimum, maximum, median and interquartile range; the lower the distance, the better the agreement. Corrected profiles with a significantly lower distance than the corresponding uncorrected profiles (unilateral exact Mann–Whitney test, *P* < 0.05) are marked with a star. Click here for file

Additional file 5: Figure S4 *P* values from bilateral Mann–Whitney tests performed on the Berger-Parker index from corrected and non-corrected twin microbiomes in function of the number of samples used. The samples were sorted by increasing Berger-Parker difference. Click here for file

Additional file 6: Figure S5 Enterotype classification of human gut microbiomes of a twin cohort at the genus level. **(A)** Before correction, **(B)** after phylogenetic-level correction, and **(C)** taxa driving the variance between samples. Click here for file

Additional file 7: Figure S6 Ordination plots illustrating how a large bias can make the correction of another bias appear ineffective. **(A)** Before and **(B)** after correction. For example, the large bias could be DNA extraction, and the smaller one gene copy number variation between species. Click here for file

Additional file 8: Figure S7 Genus-level heatmap of the human gut microbiomes before and after gene copy number (GCN) correction. Non-corrected and corrected profiles represent the average of the 280 samples. Numbers indicate the GCN of the various taxa identified in the samples and bolded text emphasizes abundant taxa (over 5% in the non-corrected data). Click here for file
