# Supplementary figures and images for: CopyRighter: a rapid tool for improving the accuracy of microbial community profiles through lineage-specific gene copy number correction
Source: Microbiome. 2014 Apr 7;2:11. doi: 10.1186/2049-2618-2-11 (PMC4021573; doi:10.1186/2049-2618-2-11)

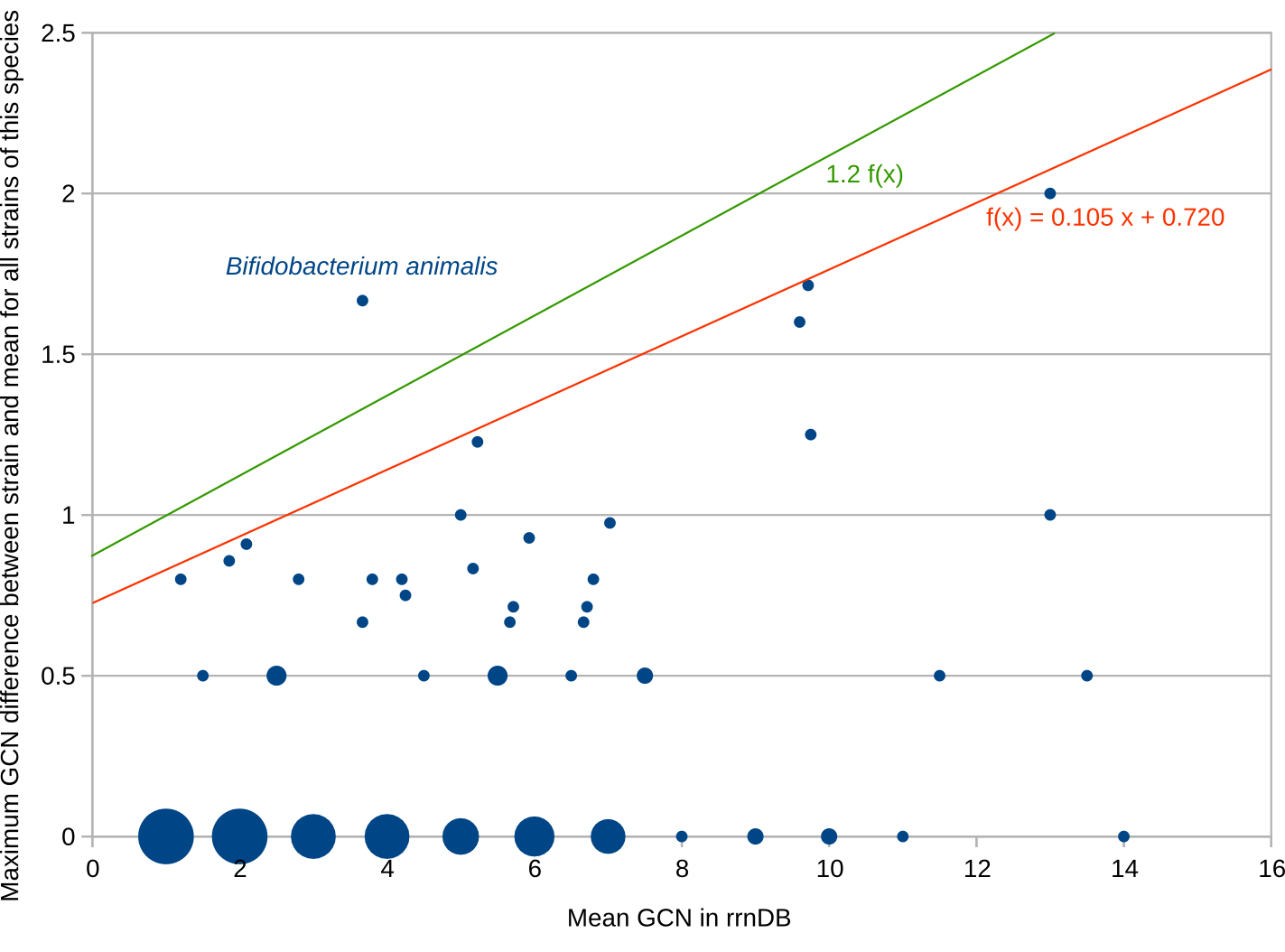

Supplement: Additional file 1: Figure S1 — Variation in gene copy number between strains of the same species in the Ribosomal RNA Database. The size of the bubbles indicates the number of species represented, from 1 to 23. [file 2049-2618-2-11-S1.png]

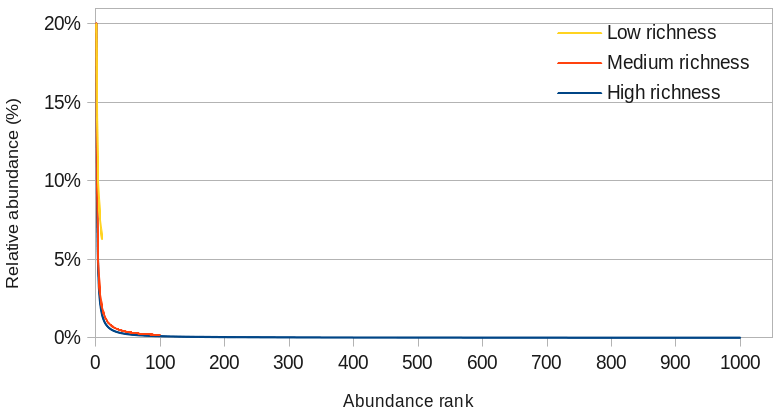

Supplement: Additional file 2: Figure S2 — Rank-abundance plot of the low, medium and high richness in silico mock communities generated with Grinder. [file 2049-2618-2-11-S2.png]

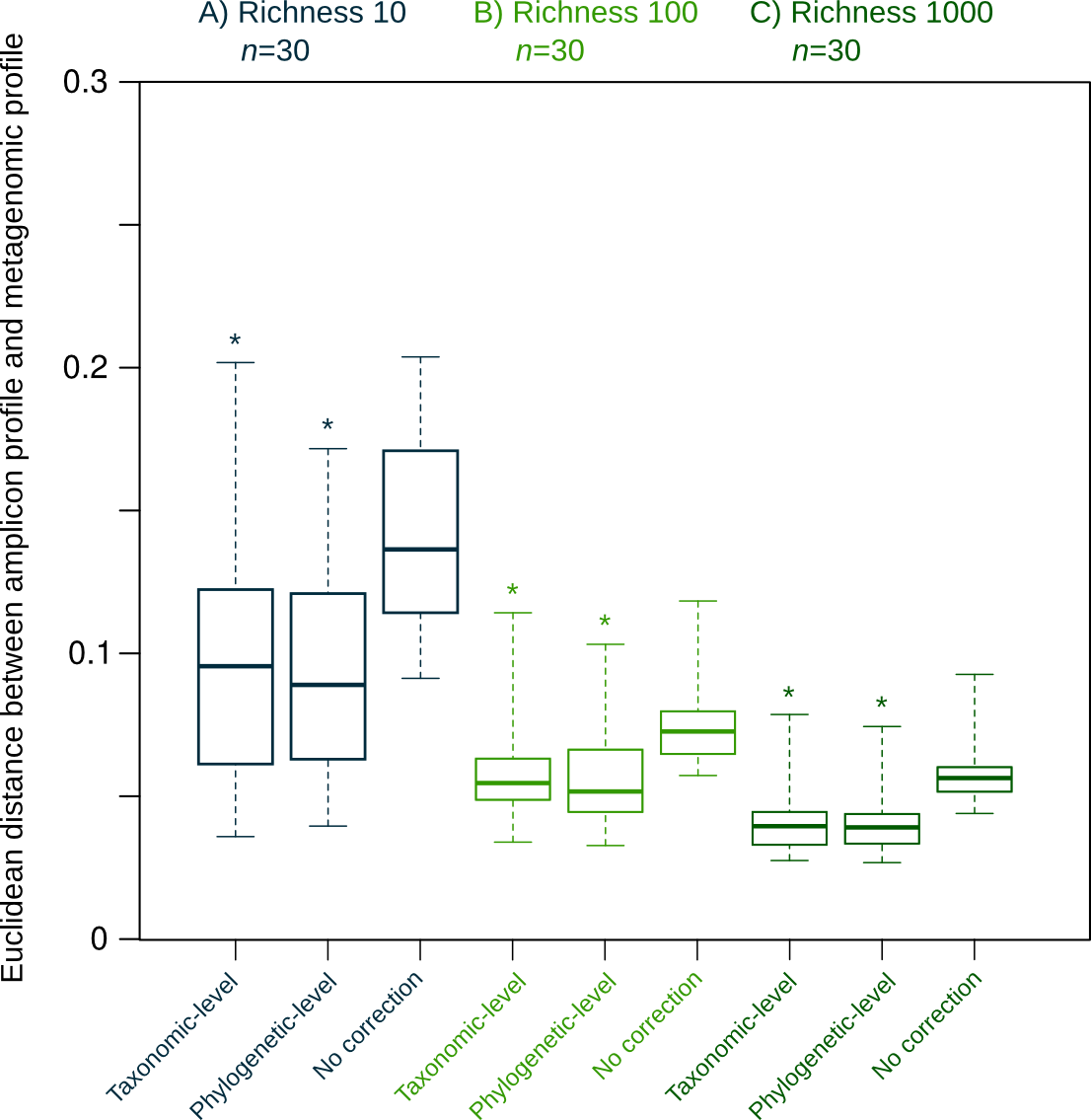

Supplement: Additional file 4: Figure S3 — Boxplot of the agreement between in silico 16S rRNA gene amplicon and metagenomic mock datasets with and without Copyrighter correction. The boxes represent the minimum, maximum, median and interquartile range; the lower the distance, the better the agreement. Corrected profiles with a significantly lower distance than the corresponding uncorrected profiles (unilateral exact Mann–Whitney test, P < 0.05) are marked with a star. [file 2049-2618-2-11-S4.png]

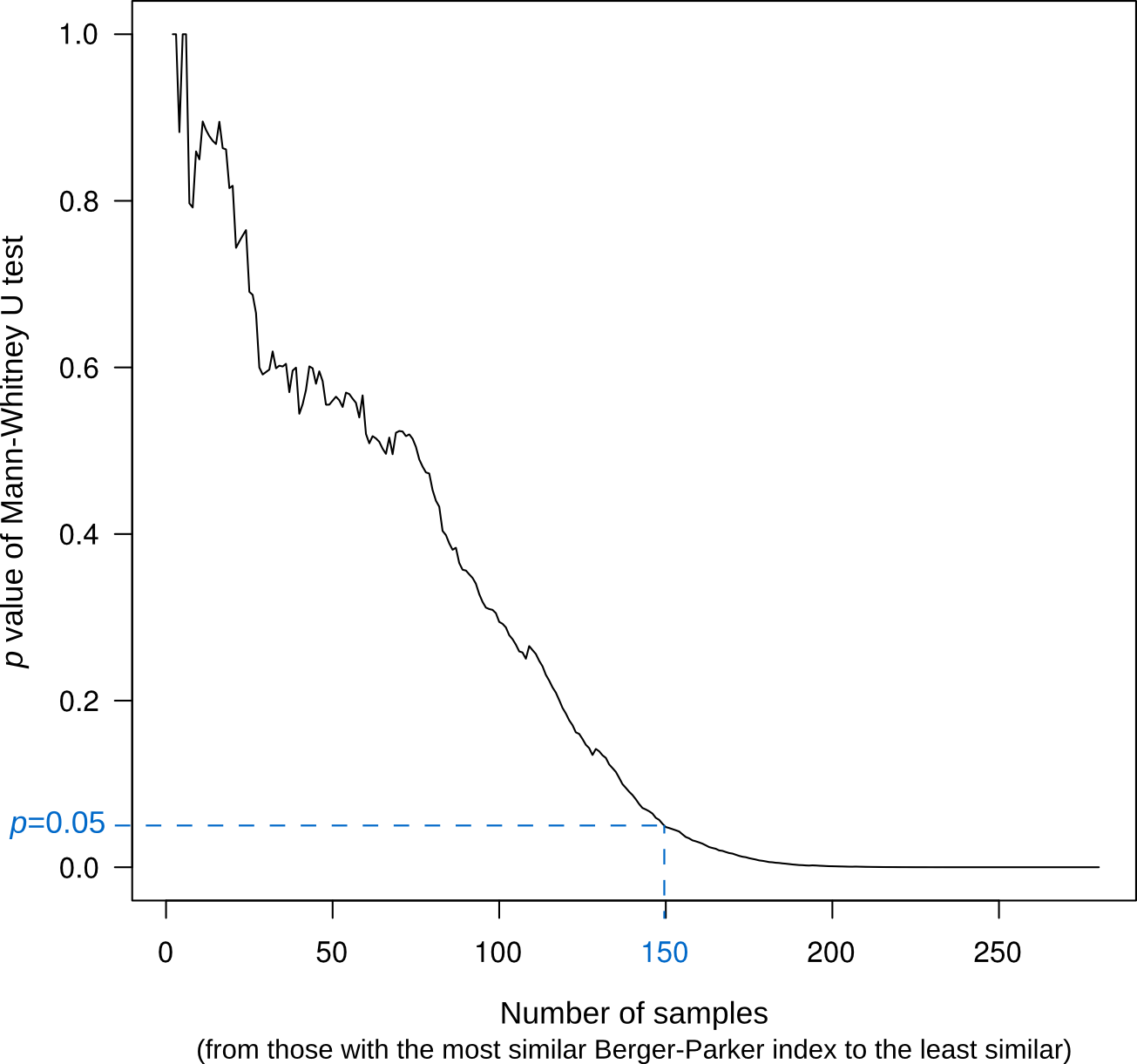

Supplement: Additional file 5: Figure S4 — P values from bilateral Mann–Whitney tests performed on the Berger-Parker index from corrected and non-corrected twin microbiomes in function of the number of samples used. The samples were sorted by increasing Berger-Parker difference. [file 2049-2618-2-11-S5.png]

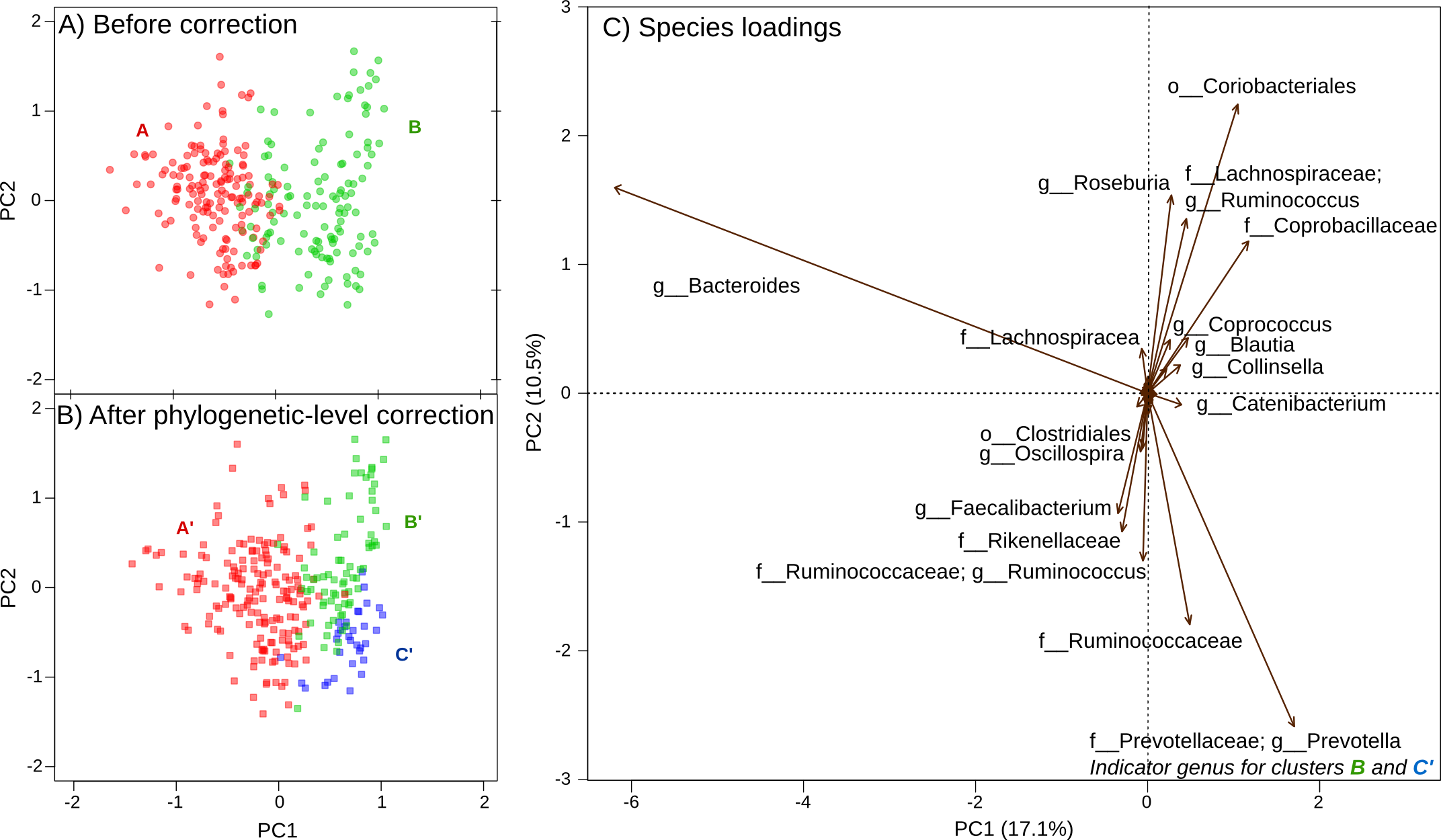

Supplement: Additional file 6: Figure S5 — Enterotype classification of human gut microbiomes of a twin cohort at the genus level. (A) Before correction, (B) after phylogenetic-level correction, and (C) taxa driving the variance between samples. [file 2049-2618-2-11-S6.png]

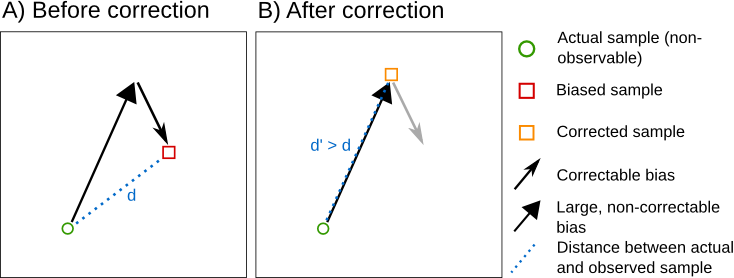

Supplement: Additional file 7: Figure S6 — Ordination plots illustrating how a large bias can make the correction of another bias appear ineffective. (A) Before and (B) after correction. For example, the large bias could be DNA extraction, and the smaller one gene copy number variation between species. [file 2049-2618-2-11-S7.png]

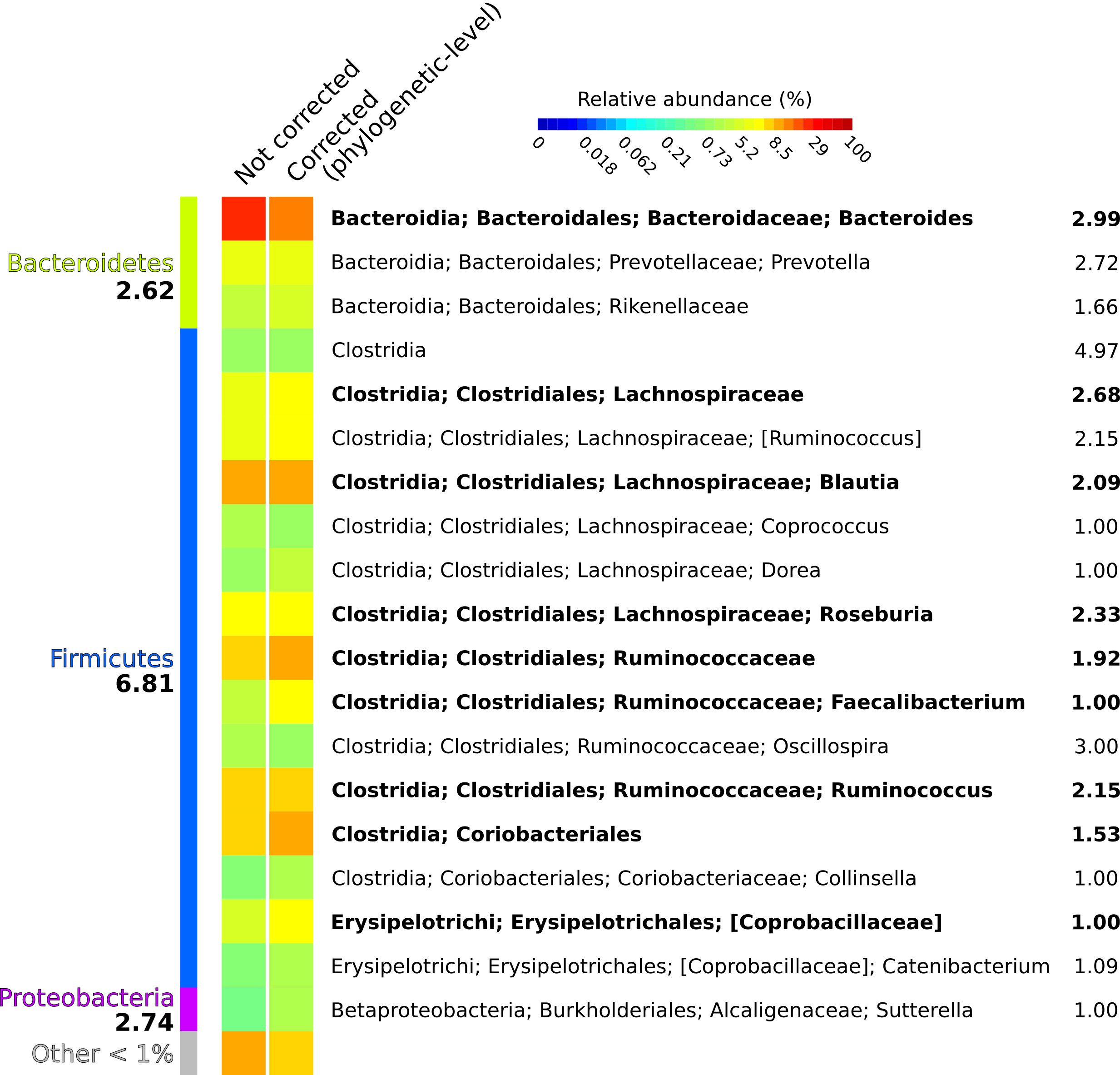

Supplement: Additional file 8: Figure S7 — Genus-level heatmap of the human gut microbiomes before and after gene copy number (GCN) correction. Non-corrected and corrected profiles represent the average of the 280 samples. Numbers indicate the GCN of the various taxa identified in the samples and bolded text emphasizes abundant taxa (over 5% in the non-corrected data). [file 2049-2618-2-11-S8.png]
