# Supplementary material for: CopyRighter: a rapid tool for improving the accuracy of microbial community profiles through lineage-specific gene copy number correction
Source: Microbiome. 2014 Apr 7;2:11. doi: 10.1186/2049-2618-2-11 (PMC4021573; doi:10.1186/2049-2618-2-11)
Supplement: Additional file 3 — Supplementary protocol: operating and sampling anaerobic digesters. [file 2049-2618-2-11-S3.pdf]

Supplementary protocol: Operating and sampling anaerobic digesters

Inoculum

Two replicate anaerobic digesters named AD1 and AD3 were set-up in parallel and seeded with the same diverse inoculum (20% v/v) which consisted of samples taken from eight different anaerobic locations: six well-functioning engineered systems (three mesophilic ADs, thermophilic AD, Upflow Anaerobic Sludge Bioreactor (UASB) and anaerobic lagoon), and two natural environments (rumen and lake sediment).

Reactor set-up and operation

The replicate anaerobic digesters (2 L working volume) were run as semi-continuous completely mixed reactors with a hydraulic and sludge retention time (H/SRT) of 10 days. The temperature was held at 37°C (± 1°C) and pH was maintained at 7 by adding 1 M NaOH solution. Alpha cellulose (Sigma Aldrich, NSW Australia) was used as a feedstock to reduce the substrate complexity and minimise influence of microorganisms otherwise entering the system through a non-sterile feed. The sterile medium consisted of 3 g L^{-1} Na_2HPO_4, 1 g L^{-1} NH_4Cl, 0.5 g L^{-1} NaCl, 0.2465 g L^{-1} $MgSO_4.7H_2O$, 1.5 g L^{-1} KH_2PO_4, 14.7 mg L^{-1} $CaCl_2$, 2.6 g L^{-1} $NaHCO_3$, 0.5 g L^{-1} $C_3H_7NO_2S$, 0.25 g L^{-1} $Na_2S.9H_2O$, and 1 mL of trace solution containing 1.5 g L^{-1} $FeSO_4.7H_2O$, 0.15 g L^{-1} H_3BO_3, 0.03g L^{-1} $CuSO_4.5H_2O$, 0.18 g L^{-1} KI, 0.12 g L^{-1} $MnCl_2.4H_2O$, 0.06 g L^{-1} $Na_2Mo_4.2H_2O$, 0.12 g L^{-1} $ZnSO_4.7H_2O$, 0.15 g L^{-1} $CoCl_2.6H_2O$, 10 g L^{-1} EDTA and 23 mg L^{-1} $NiCl_2.6H_2O$ [1]. The medium was sparged with N_2 and then autoclaved at 121°C for 60 min for oxygen removal and sterilisation, respectively. The pH was adjusted to ~7.2 by addition

of HCl (37 vol%). The reactors were fed semi-continuously with alpha cellulose (5 g cellulose L^{-1}_{medium}) at intervals of six hours, i.e. four times daily. During these feed events, approximately 50 mL of feed was pumped through the systems simultaneously using multi-head peristaltic pumps (John Morris Scientific, QLD Australia). This resulted in an organic loading rate (OLR) of 0.5 g alpha cellulose $L^{-1}_{reactor\ volume}$ d^{-1}. Semi-continuous feeding mode was switched off between Days 12 and 24 (until a sufficient decrease in VFA concentration was observed) in order to minimize wash-out of slow-growing microorganisms and to allow the biomass in the reactors to increase.

DNA extraction and 16S rRNA gene amplicon sequencing

Samples for DNA extraction were taken at day 27 and 362, snap-frozen in liquid nitrogen and stored at -80 °C. DNA extractions were performed using FastDNA Spin kits for Soil (MPBio, US) according to the manufacturer's instructions. DNA quality was assessed using gel electrophoresis (1% agarose) and DNA concentrations were measured using Quant-iT dsDNA BR Assay kits and a Qubit fluorometer (Invitrogen, US).

Genomic DNA was extracted from samples taken from each reactor at 14 time points and 16S rRNA genes were amplified as described previously [2]. Briefly, PCR reactions (50 µL) were prepared with 20 ng of template DNA, 5 µL 10x buffer, 1 µL dNTP mix (10 mM each), 4 µL 25 mM MgCl, 1 µL forward primer (10 mM), 1 µL reverse primer (10 mM), 0.2 µL *Taq* polymerase and 1.5 µL BSA (Invitrogen, US) and 1µL (10 mM) of each of the universal primers targeting the V6-V8 region of the bacterial and archaeal 16S rRNA gene [3]: 926F (5'-AAACTYAAAKGAATTGRCGG-3') and 1392R (5'-ACGGGCGGTGTGTRC-3') modified on the 5' end to contain 454 sequencing adaptor sequences. The reverse primer also contained a

5-6 base sample-specific barcode sequence. The PCR program included one cycle at 95°C for 3 min, followed by 30 cycles at 95°C for 30 s, 55°C for 30 s and 75°C for 30 s, and then a final extension at 74°C for 10 min. Post amplification, amplicons were pooled and sequenced using the Roche 454 GS-FLX Titanium platform at the Australian Centre for Ecogenomics. Sequences were submitted to the NCBI Short Read Archive with accession number SRR1145444.

Real-time quantitative PCR

Total bacterial and archaeal biomass was estimated using real-time PCR for the two reactors at the same two time points subjected to 16S rRNA gene amplicon sequencing. The primers 1406F (5‘-GYACWCACCGCCCGT-3’) and 1525R (5’-AAGGAGGTGWTCCARCC-3’) were used to amplify bacterial and archaeal 16S rRNA genes. For inhibition control, the rpsL F (5’-GTAAAGTATGCCGTGTTCGT-3’) and rpsL R (5’-AGCCTGCTTACGGTCTTTA-3’) primer set was used, which is specific for *E. coli* DH10B rRNA. Two dilutions (1/100, 1/1000) were made of the microbial template DNA and run in parallel with an inhibition control test using *E. coli* DH10B genomic DNA. The PCR reaction was set up using 5 µL 2x SYBR Green/AmpliTaq Gold DNA Polymerase mix (Life Technologies), 4 µL template DNA and 1 µM primer mix (0.4 µM 1406F/1525R, 0.2 µM rpsL F/R) and each sample was run in triplicate. The PCR program included one cycle at 95°C for 10 min, followed by 40 cycles of 95°C for 15 s and 60°C for 1 min. A melt curve was produced by running one cycle at 95°C for 2 min and a final cycle at 60°C for 15 s. The cycle threshold (Ct) values were recorded and analysed using ABI SDS 2.4.1 software.

References

1. Dennis PG, Guo K, Imelfort M, Jensen P, Tyson GW, Rabaey K: **Spatial uniformity of microbial diversity in a continuous bioelectrochemical system**. *Bioresour Technol* 2013, **129**:599–605.

2. Cayford BI, Dennis PG, Keller J, Tyson GW, Bond PL: **High-throughput amplicon sequencing reveals distinct communities within a corroding concrete sewer system**. *Appl Environ Microbiol* 2012, **78**:7160–7162.

3. Engelbrektson A, Kunin V, Wrighton KC, Zvenigorodsky N, Chen F, Ochman H, Hugenholtz P: **Experimental factors affecting PCR-based estimates of microbial species richness and evenness**. *ISME J* 2010, **4**:642–647.
